# Strategies for Implementing GlobalConsent to Prevent Sexual Violence in University Men (SCALE): Study Protocol for a National Implementation Trial

**DOI:** 10.21203/rs.3.rs-4745916/v1

**Published:** 2024-09-10

**Authors:** Kathryn M Yount, Daniel Whitaker, Xiangming Fang, Quach Thu Trang, Meghan Macaulay, Minh Tran Hung

**Affiliations:** Emory University Rollins School of Public Health; Georgia State University School of Public Health; Georgia State University School of Public Health; Center for Creative Initiatives in Health and Population; Emory University Rollins School of Public Health; Center for Creative Initiatives in Health and Population

**Keywords:** cost effectiveness, educational entertainment, GlobalConsent, implementation trial, low- and middle-income country, primary prevention, sexual violence, Vietnam

## Abstract

**Background:**

Globally, women 15–24 years are at heightened risk of sexual violence victimization, a risk factor for adverse mental, physical, and behavioral health outcomes. Sexual violence is common at universities and most often perpetrated by men, yet few evidence-based prevention strategies targeting men have been tested in low- and middle-income countries. GlobalConsent is a six-module, web-based educational program adapted from an efficacious U.S.-based program. Nine months post-treatment in a randomized trial in Vietnam, GlobalConsent reduced men’s sexually violent behavior (Odds Ratio [OR] = 0.71, 95%CI 0.50–1.00) and increased prosocial intervening behavior (OR = 1.51, 1.00–2.28) relative to an attention-control. Evidence regarding optimal implementation strategies for scale up is needed.

**Methods:**

We will randomize six medical universities in North, Central, and South Vietnam to deliver GlobalConsent using two different packages of implementation strategies that vary in intensity. Higher-intensity strategies will include greater 1) pre- and post-implementation engagement with university leaders and faculty, and 2) greater pre-implementation outreach, follow-up, and incentives for students to promote engagement and completion of GlobalConsent. Higher intensity universities will receive additional training and support for their added activities. We will compare implementation drivers and outcomes, intervention effectiveness, and cost-effectiveness across the two implementation bundles. Our mixed-methods comparative interrupted time-series design includes 1) qualitative interviews and quantitative surveys with university leaders and implementation teams to assess implementation barriers and facilitators; 2) repeated surveys with leaders and faculty, implementation teams, and male students to assess multilevel implementation drivers and outcomes, 3) repeated surveys with male students to assess behavioral outcomes (sexual violence and intervening behavior) and mediating variables (knowledge, attitudes, affect, and capacities), and 4) time diaries and cost tracking to assess cost effectiveness of the two implementation-strategies bundles.

**Discussion:**

This project is the first to assess packages of implementation strategies to deliver an efficacious web-based sexual-violence-prevention program for undergraduate men across all regions of Vietnam and synergizes with a violence-prevention training initiative (D43TW012188). This approach will produce rigorous evidence about how to disseminate GlobalConsent nationally, which holds promise to reduce gender-based health inequities linked to sexual violence as GlobalConsent is brought to scale.

**Trial registration:**

NCT06443541. Retrospectively registered with clinicaltrials.gov.

## Introduction

### Background and rationale {6a}

#### Sexual violence is prevalent in adolescence and heightens the risk of harmful long-term health effects.

Sexual violence includes any sexual act committed against a person without freely given consent [1]. All genders may experience sexual violence, but sexual violence more often burdens women than men globally [2, 3], and men most often perpetrate such violence [4, 5] Adolescence is a period of vulnerability to sexual violence [6], with about one in five college women in the US experiencing a campus sexual assault [7], and 91% of victims being women [8] Less is known about rates of sexual violence on college campuses in LMICs, but estimates from large, multi-country surveys confirm that young men’s reported sexually violent behavior [9, 10] and young women’s reported sexual violence victimization [6] are high, including in Asia/Pacific. In Vietnam [11], from 2010 to 2019, women’s reports of lifetime sexual violence by a partner increased (10% to 13%), especially in women 18–24 years (5% to 14%). Such trends may reflect changing exposure and more openness to discuss sex and sexual violence. Also, nearly one in ten women (9%) report non-partner sexual violence since age 15, mostly perpetrated by non-family male acquaintances, co-workers, or strangers. Young women who are victims of sexual violence are at heightened risk of acute and chronic mental and physical health conditions [12].

#### Evidence-based sexual-violence prevention programs tailored to men are limited.

Several reviews since 2016 confirm that interventions to prevent sexual violence in young men are rare, especially in LMICs, do not follow best practices for behavioral change, and yield mixed results. A 2017 review of reviews identified few interventions with adolescents focused on boys or young men in LMICs [13]. A subsequent review of 44 bystander intervention studies in North America found that, most often, programs are conducted in college populations (75%) and mixed-gender groups (56%) and involve a single (75%) in-person (68%) presentation and discussion (54%) of less than two hours [14]. Relatively few of these interventions were tailored to men (27%) or involved technology-based delivery using the web (11%), media (36%), or social media (7%) [14]. Study designs also tended to be weak, often involving modest sample sizes (mean 536) of majority-white participants (45%), high attrition (mean 36%), non-randomized controlled designs (62%), infrequent follow-up beyond six months (11%), and infrequent measurement of behavioral outcomes (34%) [14]. A third systematic review [15] focused on interventions to prevent intimate-partner, dating, and sexual violence in men and boys, did find that most of the nine included studies used cluster-randomized designs and evaluated multisession programs delivered in groups to undergraduates; yet, most studies were US-based and only one program reduced men’s self-reported sexually violent behavior [15]. A fourth review, focused on intervention studies to change hegemonic masculinities, found that eight of the 10 included studies were conducted in the US or Africa, only one (in the US) was web-based (but not delivered to a mobile device), and impacts on sexually violent behavior were mixed [16]. Finally, one review of 31 mHealth interventions to address partner violence found that mobile-phone platforms were acceptable, but that victim response of women was the focus over behavioral prevention with men, and evidence of efficacy was limited [17]. Thus, especially in Low- and Middle-Income Countries (LMICs), sexual-violence prevention programs tailored to men are rare, and theoretically grounded, web-based sexual-violence prevention with men has not been implemented at scale.

#### Effective sexual-violence prevention programming for men requires a cross-cultural theory of change.

**Figure 1.**
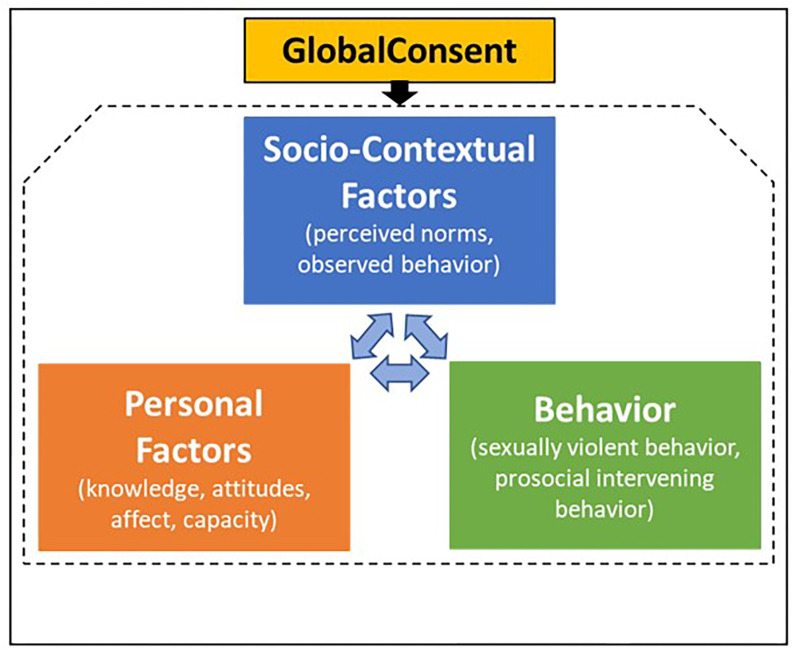
Cross-Cultural Theory of Change: Effects of GlobalConsent on Sexually Violent Behavior and Prosocial Intervening Behavior

Given the limitations of prior prevention interventions tailored to men and expanding needs in LMICs, our team’s long-term research agenda has been to develop, test, and scale an efficacious sexual-violence prevention program for young men in LMICs. To do so, our team has been guided by an integrated theory of change, drawing on social cognitive theory [18], social norms theory [19], and the bystander education model [20] (Figure 1). Social cognitive theory posits that behavior is influenced by and influences socio-contextual factors and personal factors, in a dynamic known as reciprocal determinism. Social norms are important socio-contextual factors that may promote sexually violent behavior through men’s perceptions or misperceptions of socially expected behavior. These expectations may be communicated through the media or socialization processes in families, peer networks, or institutional settings. Personal factors, including cognitions, attitudes, affect, and biological events, interact with perceived or misperceived social norms, by countering or reinforcing them. For example, a young man with more knowledge about sexual violence may be able to counter perceived messaging of sexual violence as normal, but a young man with less knowledge about sexual violence may be unable to counter such messaging. Finally, sexually violent behavior is a manifestation and perpetuation of perceived or misperceived social norms about sexual violence; whereas, prosocial intervening behavior conveys the act of intervening as normal and sexually violent behavior as abnormal. Thus, one man’s behaviors can reinforce or modify social norms about sexual violence, and in turn, the behaviors of peer witnesses. The nature of and interactions between socio-contextual factors, personal factors, and sexually violent behavior may vary across societies and cultures, but the interplay of these factors is thought to be widespread [21].

Thus, the program we adapted and tested in Vietnam, GlobalConsent, was designed to disrupt the reinforcing interplay between 1) pro-violence socio-contextual factors including perceived or misperceived social norms that sexually violent behavior is normal, 2) pro-violence personal cognitions, attitudes, and affect, and 3) weak social norms of intervening behavior. By disrupting this interplay, GlobalConsent was able to reduce sexually violent behavior and to increase prosocial intervening behavior among men attending two universities in Vietnam. In sum, the scale of sexual violence in adolescence, its long-term health effects, the strong theoretical premise of GlobalConsent, its efficacy, and the need for national programming in Vietnam motivate our team’s next step—to test two strategies to implement GlobalConsent at scale. This step is unprecedented in the global violence-prevention field and paves a path for national uptake.

#### Implementation strategies may need to be bundled for public health impact.

A key question now is how best to implement GlobalConsent to achieve broad public health impact. Research has confirmed that strong implementation is needed to continue to see outcomes obtained in clinical trials [22, 23], and that very simple implementation methods often do not yield implementation, implementation with fidelity, or sustained implementation [23]. Though the field of implementation science still is young, several theoretical models specify 1) the stages and processes of implementation, and 2) multilevel influences that act as barriers or facilitators of implementation. For example, the Reach-Effectiveness-Adoption-Implementation-Maintenance (RE-AIM) model [24] specifies reach, effectiveness, adoption, implementation, and maintenance as multilevel outcomes that drive public health impact. An expanded version of RE-AIM identifies contextual factors in the outer and inner (organizational) setting related to these outcomes [25, 26]. The Evidence-based Practice Implementation in public Service sectors (EPIS) model [27] specifies stages of implementation for the adoption of an innovation, including exploration, pre-implementation, implementation, and sustainment. At a more micro level, others have identified key processes within an implementation that lead to success, including skills-based training, follow-up coaching, administrative supports, and examination of implementation and outcome data to ensure results [28]. Salient implementation strategies across organizational levels can be clustered to focus on developing interrelationships between stakeholders and engaging leadership, training and supporting those delivering the intervention, engaging potential consumers, and addressing institutional norms and infrastructure to facilitate intervention delivery [29, 30]. A key question is how to employ these strategies efficiently; strong bundled implementation strategies can be resource intensive, and understanding their incremental cost-effectiveness is crucial, especially in LMICs. We aim to compare 1) the implementation of GlobalConsent, 2) implementation drivers and outcomes, 3) effectiveness outcomes, and 4) cost-effectiveness across two bundles of implementation strategies.

### Objectives {7}

The primary objective of this study is to conduct an implementation trial of GlobalConsent in six universities across Vietnam. We will use the RE-AIM [24, 31] and Proctor et al. [32] frameworks and a mixed-methods, comparative interrupted time series (CITS) design to compare implementation; implementation drivers and outcomes; implementation effectiveness; and cost-effectiveness of lower-intensity implementation strategies (LIS) versus higher-intensity implementation strategies (HIS) to deliver GlobalConsent. Pair-matched study universities will be assigned randomly to LIS or HIS groups, and a local implementation team at each university will support the intervention (which is delivered digitally).

Implementation strategies will target multiple stakeholder groups -- university leaders, faculty, and students -- to address barriers and facilitators to implementation at multiple institutional levels. The LIS universities will deliver basic implementation strategies often used to deliver online programs at US universities [15]. The HIS universities will deliver additional strategies and at a higher intensity [30] that were identified in the GlobalConsent trial [33] and from learning-collaborative research [34] and that align with interviews [35] with influential university leaders across Vietnam. Leaders in both groups will receive pre-implementation educational outreach to address knowledge and normative institutional barriers to implement GlobalConsent. University leaders in the HIS group will receive additional outreach during and after implementation. University faculty in the HIS Group, but not the LIS Group, will be engaged around supporting the implementation. University students in both groups will receive remote invitations to take part in and reminders to complete GlobalConsent. Students in the HIS group *also* will receive from internal facilitators pre-implementation in-person outreach, incentives to increase demand, and more frequent reminders to progress and complete the program.

Regarding training and support, internal implementation teams in both groups will receive manualized pre-implementation training on how to support and deliver GlobalConsent. Implementation teams at HIS universities will receive more intensive training and support than those at LIS universities to implement the additional strategies, described above, including pre-implementation leadership training to champion GlobalConsent with internal and external stakeholders.

Our four specific aims are to:

**Aim1. Compare implementation (barriers, facilitators, modifications**) of delivering GlobalConsent in LIS versus HIS groups [36]. Separate focus group discussions with university implementation teams and the purveyor/training entity (CCIHP) will identify implementation barriers, facilitators, and modifications for HIS and LIS universities. Key informant interviews with university leaders will be used to identify organizational, internal and external policy conditions that may affect implementation. A checklist to male students will identify modifications to intended program delivery at the user level.

**Aim2. Compare implementation drivers** (e.g., institutional norms) and **outcomes** (e.g., penetration) in LIS and HIS universities. Assessments will be based on repeated surveys with university leaders, implementation teams, faculty, and male students; and administrative data on program adoption and penetration among male students. We expect implementation drivers and outcomes to be more favorable in the HIS versus the LIS group over time because of the implementation efforts.

**Aim3. Compare effectiveness outcomes** (knowledge, attitudes, affect, capacities, and behavior related to sexual violence) in the LIS and HIS groups using six-monthly surveys with male university students. We expect that men in the HIS group will report more favorable, more sustained outcomes in all domains than men in the LIS group.

**Aim4. Evaluate the cost-effectiveness** of implementing GlobalConsent in the HIS group versus the LIS group. We expect that HIS will be cost-effective relative to LIS, e.g., HIS’s additional costs will be justified by the greater impact on reducing sexually violent behavior and increasing prosocial intervening behavior when compared to LIS.

This study is the first to assess two multifaceted implementation strategies to deliver a theoretically grounded, efficacious web-based sexual-violence prevention program to male students attending six universities across Vietnam. If successful, our multidisciplinary, cross-cultural team will be the first to bring rigorous evidence to university and national leaders of the contextual effectiveness of these strategies for delivering web-based sexual-violence prevention programming to large populations of men in adolescence, a period of heightened risk for sexually violent behavior. Our choice to develop, test, and scale GlobalConsent with universities in Vietnam is strategic, given the scale of sexual violence among young people, expanding rates of university attendance [37–39], and the openness of several university leaders to efficacious programming about sexual violence. Our choice to engage universities across all regions of Vietnam provides a novel test of these implementation strategies in different structural and sociopolitical environments, with promise to advance sexual-violence prevention policies in university systems at regional and national levels. Evidence for the effectiveness and incremental cost-effectiveness of these implementation strategies across regions will pave the way for GlobalConsent to address an important, gendered risk factor for chronic mental, physical, and behavioral health conditions over the life course. Thus, by providing novel evidence about how best to bring GlobalConsent to scale nationally, our team has the potential to reduce gender-related health inequities and to improve quality of life by averting acts of sexual violence that may lead to chronic health conditions over the life course among victims. By partnering with universities engaged in CONVERGE, an on-going violence-prevention training program in Vietnam (D43TW012188), these innovations will be achieved through synergistic investments to strengthen local capacity for implementation research, data harmonization, and stakeholder engagement to manage and to prevent sexually violent behavior in young people.

### Trial design {8}

The present study is a mixed-methods, comparative interrupted-time-series (CITS) study [40, 41] to compare the implementation metrics, drivers and outcomes, effectiveness, and cost-effectiveness of two bundled implementation strategies to deliver GlobalConsent to men attending six pair-matched universities in North, Central, and South Vietnam. The Framework for Reporting Adaptations and Modifications-Expanded (FRAME) [36] guides the Aim 1 implementation assessments. The RE-AIM [24, 25, 42] and Proctor et al. [32] frameworks guide the Aim 2 implementation drivers and outcomes and Aim 3 implementation effectiveness assessments. A micro-costing approach guides the Aim 4 cost-effectiveness assessment [43, 44].

## Methods: Participants, interventions and outcomes

### Study setting {9}

Table 1 provides details on the six universities taking part in this proposed study, including location, year founded, programs of study, and faculty/student population sizes. Partnering universities have experience collaborating on studies supported by major funders, including the National Institutes of Health (NIH), World Health Organization (WHO), United States Agency for International Development (USAID), and the President’s Emergency Plan for AIDS Relief (PEPFAR)/Substance Abuse and Mental Health Services (SAMHSA). Several universities are partners in an on-going science leadership program in violence prevention [45]. Several also have partnered with the primary institutions (Emory University and the Center for Creative Initiatives on Health and Population [CCIHP]) on prior projects, and thus, have experience with the kind of collaboration proposed here. Lastly, across universities, there is substantial interest in sexual violence and intersecting health-related topics, including HIV prevention, sexual and reproductive health, general violence prevention, and preventive medicine. These interests and strong letters of support bode well for ensuring a strong commitment to the proposed project.

### Eligibility criteria {10}

#### University leaders (Aim 1).

We will sample university leaders purposively to identify those most knowledgeable of and critical to the implementation of GlobalConsent [46, 47]. To the extent possible, leaders will be matched on position and rank across LIS and HIS groups (e.g., Dean/Vice Dean and Department Head/Deputy Head). If necessary to achieve saturation, we will use snowball sampling based on recommendations from interviewed stakeholders to identify additional leaders to serve as key informants [48].

#### Implementation team members (Aims 1, 2, and 4).

Implementation team members will be sampled purposively to identify those with the most relevant expertise for the implementation of GlobalConsent. To the extent possible, implementation team members will be matched on position and rank (e.g., lecturer and/or staff by rank) across LIS and HIS groups.

#### University faculty (Aim 2).

All fulltime, permanent lecturers who are currently working (not on extended leave) and not in the leader or implementation team samples at the time of the baseline faculty survey will be eligible to participate. The list of eligible lecturers will be refreshed before each survey wave to ensure that all eligible lecturers are included at each wave.

#### First-year male students (Aims 1 and 3).

Eligible student participants will be male (sex assigned at birth), 18–24 years old at first contact, self-identified as heterosexual or bisexual (are attracted to women) and matriculating into the study universities in project year 2.

### Who will take informed consent? {26a}

#### Key informant interviews (KIIs) and focus group discussions (FGDs) and reflections (Aim 1).

Researchers at CCIHP who will conduct the key informant interviews and who will facilitate the focus group discussions will obtain consent from each participant. Because written informed consent is not considered suitable for this setting, local interviewers and group facilitators will digitally record verbal informed consent with a witness before starting the qualitative interviews and group discussions. Informed consent for the qualitative research will require a clear understanding of the study’s purpose; voluntariness, nature, extent and duration of participation; procedures to ensure confidentiality; and right to not answer questions or to withdraw from the study at any time. Participants will be informed that, with permission, interviews will be digitally recorded, and interviewers will keep field diaries of their observations and experiences with participants. All qualitative data collection will be conducted in private settings at the study sites, where the interviewers and facilitators will provide more detail about the exact nature of the study, procedures, and any expected risks and benefits to eligible participants. If necessary, interviews and focus group discussions may be conducted via HIPAA-compliant video-conferencing software. Participants will be informed that 1) digital-recordings and fully de-identified Vietnamese and English transcripts will be uploaded to separate folders on a HIPAA-compliant, secure network drive maintained by Emory University, with access limited to the study team for a specified duration before being destroyed; 2) that analysis of the transcripts will take place on a secure, password protected computer in a private space that can be locked; 3) that all participants will be compensated for their time and will be offered refreshments (for in-person interviews and group discussions). Participants also will be informed that the study team will recontact them at a later point in the study for follow-up interviews and/or focus group discussions (**Appendix**).

#### Online quantitative surveys (Aims 2–3).

Eligible participants in the quantitative portions of the study will read an informed consent form provided in an online REDCap survey [49]. Eligible participants will indicate with check boxes that they have read each paragraph in the consent form and will be provided with a phone number to call if they have any questions that a non-study team member can answer. After all paragraphs are checked, the eligible participant will be invited to provide a response to confirm their consent or non-consent to participate in the study. Informed consent will be obtained before participants will be allowed to view and to participate in the online REDCap survey for which they are eligible (**Appendix**).

#### Costing surveys (Aim 4).

CCIHP staff (including external facilitators and administrative staff involved in GlobalConsent) and university GlobalConsent implementation team members will complete costing forms regularly, either monthly or weekly, depending on the implementation phase. These individuals will receive a consent form to sign, indicating their agreement to participate. The consent form will include an information sheet, a statement confirming that they have read and understand the information sheet, a statement agreeing to participate in the survey, and contact information for a person who can answer any questions or concerns. The information sheet will cover the purpose of the costing surveys, how the data will be collected, used, and stored, the voluntary nature of participation, an assurance of confidentiality, risks and benefits of participation, and procedures to manage any risks (**Appendix**).

### Additional consent provisions for collection and use of participant data and biological specimens {26b}

There are no additional consent provisions for the collection and use of participant data in this study. No biological specimens are being collected.

## Interventions

### Explanation for the choice of comparators {6b}

A local implementation team at each participating university will deliver the GlobalConsent intervention. This study will vary the implementation strategies that are delivered, with some universities using lower-intensity implementation strategies (LIS) and other universities using higher-intensity implementation strategies (HIS). Implementation strategies in the LIS group model standard approaches to deliver online sexual violence primary prevention programs in universities in the U.S. Implementation strategies in the HIS group were selected from those used in the GlobalConsent efficacy trial [33, 50, 51], from the literature on learning collaboratives [34], and to address barriers and facilitators to program implementation that are common to university settings in Vietnam [35] (Table 2). Nomenclature for specific implementation strategies follows the Expert Recommendations for Implementing Change (ERIC) project [30]. The LIS and HIS implementation strategies are discussed by cluster and organizational level, where key stakeholders were identified to understand the implementation process (Aim 1), drivers and outcomes (Aim 2), and effectiveness (Aim 3).

#### Develop interrelationships between stakeholders and engage university leaders.

Given the demonstrated efficacy of GlobalConsent and the need for high-level institutional commitment for successful implementation, some strategies with university leaders [52] (Deans, Vice Deans, Department heads, Deputy heads) are common to both groups, and the HIS group will receive additional strategies (Table 2). Implementation strategies in both IS groups include prework to obtain formal commitments [30, 34]; passive external web-support with educational materials [34]; *and* pre-implementation educational outreach [34]. Prework, led by CCIHP, involves invitations to each university to take part, a written summary of the proposed project, site-specific dialogue, and written commitments to participate. External web-based support typically is available to US-universities delivering online sexual-violence prevention programs [53, 54]. Our Vietnam-based website will include links to the open-access GlobalConsent study protocol and impact assessments [33, 50, 51]; short videos providing an overview of sexual violence and GlobalConsent in Vietnam and explaining findings in lay terms; one-page briefs with overviews of GlobalConsent and sexual violence among young people in Vietnam; one-page briefs about these findings and sexual violence among young people in Vietnam generally; and answers to frequently asked questions. The two-hour pre-implementation educational outreach led by CCIHP will address misinformation about sexual violence as uncommon, the normative climate for evidence-based prevention, and a description of the GlobalConsent program.

#### Engage potential consumers (university-student users of GlobalConsent).

Internal facilitators in both IS groups will prepare students to become active consumers of GlobalConsent with an email introduction to the program, process and schedule for delivery, procedures for data collection, and consent to take part.

#### Train and support internal implementation facilitators and teams.

Internal implementation facilitators and teams in both IS groups will receive passive external web-support with educational materials and three days of in-person pre-implementation manualized educational outreach on how to deliver GlobalConsent to eligible undergraduate men at their university (Table 3). The three days of in-person training will cover procedures about how to: maintain records on program adoption and penetration among students; identify and invite eligible students to complete an online informed consent, and if completed, a series of short surveys; and send text and email reminders to complete each module within two weeks.

### Intervention description {11a}

Implementation strategies in the HIS group include *additional* strategies (1) to develop inter-relationships between stakeholders and to engage university leadership, and (2) to engage potential consumers (student users) of GlobalConsent (Table 2). CCIHP will provide additional training and support to the implementation team facilitators and members who are delivering GlobalConsent, in service of carrying out the intervention (Table 3).

#### Develop interrelationships between stakeholders and engage university leaders.

Additional implementation strategies to develop interrelationships and to engage university leaders only in the HIS group will include internal-facilitator efforts to inform local opinion leaders [34] about implementation progress via regular emails to university leaders. Additional efforts to inform local opinion leaders will involve meetings with the university faculty (organized and led by university implementation teams) about sexual violence as a problem and prevention with GlobalConsent. Post-implementation educational outreach will entail a one-hour webinar jointly organized by CCIHP and internal implementation teams sharing anonymized findings by IS group and discussing plans to sustain GlobalConsent with future cohorts of university men.

#### Engage potential consumers (university-student users of GlobalConsent).

Students only in the HIS group additionally will receive: educational outreach in a pre-implementation in-person orientation to GlobalConsent covering similar topics and three monthly one-hour learning sessions during implementation in which technical questions about program access or progression can be addressed; more intensive intervention to enhance adherence with more frequent email/SMS completion reminders (weekly versus every two weeks for 12 weeks); and demand generation with an option to enter a lottery to win prizes upon program completion.

#### Train and support internal implementation facilitators and teams.

Internal implementation teams and facilitators only in the HIS group additionally will receive leadership training before implementation and external support and technical assistance (TA) during implementation. The two-day leadership training will cover skills needed to champion GlobalConsent with diverse internal stakeholder groups. Topics will cover leadership styles, managing teams, influence without authority, managing conflict, emotional intelligence, negotiation, and leading institutional change. The leadership training also will cover effective ways to facilitate a student orientation to GlobalConsent, facilitate faculty/staff town halls (outreach sessions) about sexual violence, and send effective communications to leaders on implementation progress (Table 3). On-going external support and TA will involve six one-hour quality-improvement team consultations with CCIHP to provide refresher training, discuss implementation progress and modifications, build peer networks, and discuss anonymized data on implementation progress for shared problem-solving. Consultation sessions will be recorded and later coded as part of data collection activities.

### Criteria for discontinuing or modifying allocated interventions {11b}

CCIHP will encourage adherence to the implementation plan of the LIS and HIS groups in response to any questions that are posted to the GlobalConsent website regarding deviations to the implementation plan. CCIHP also will encourage adherence to the implementation plan of the HIS group in its regular consultative meetings, when it provides technical support. The study team will conduct regular focus group discussions with implementation teams to monitor modifications to implementation strategies that LIS and HIS implementation teams may apply (Aim 1). The decision to discontinue a student’s participation in GlobalConsent will be made based on an adverse events protocol that is described elsewhere in this study protocol.

### Strategies to improve adherence to interventions {11c}

To improve implementation-team adherence to the LIS and HIS implementation strategies protocols, Emory and CCIHP will establish deliverable-based contracts with each university clarifying the terms of reference (TOR) and payment schedule for mutually agreed implementation activities. At the time of implementation team training, each team member at each participating university will assume specific roles and responsibilities, and a team supervisor will be responsible for overseeing the activities of all implementation team members. The completion of all activities will be assessed at the time that each university invoices CCIHP for its work, and payment of invoices will be based on the demonstrated completion of implementation activities in the TOR.

To support students’ adherence to the GlobalConsent program in the HIS group, the contracted IT company (with support from internal implementation team members) will send students weekly email and/or text reminders to complete each program module. In the LIS group, the contracted IT company, with support from internal implementation team members, will send students email and/or text reminders once every two weeks to complete each program module. Students will be offered to enter their unique ID into a lottery to win a small prize for completing each module. Student adherence to program participation will be monitored with a short survey after each program module and with passive monitoring by the IT company delivering the program (times module opened, time spent with module open).

### Relevant concomitant care permitted or prohibited during the trial {11d}

There is no concomitant care that is specifically permitted or prohibited during the trial.

### Provisions for post-trial care {30}

A case management protocol for post-trial care is applied to all students who participate in the GlobalConsent program and each survey wave. First, near the end of each survey, all student participants are provided a comprehensive resource list of local fee-based and non-fee-based services. This resource list is provided to all participants. Second, after providing the resource list, all participants are asked to report their level of distress (1=not at all distressed to 10=extremely distressed) and the manageability of their reported distress (0=manageable, 1=manageable with resources, or 2=not at all manageable). Any participant who reports extreme distress (=10) or “not at all manageable” distress (=2) regardless of the distress level reported will receive an emergency contact number in REDCap and will be offered the opportunity to follow-up with a professional counselor. The message will read: “Your wellness is important to us, and someone outside of the study can follow-up with you, if you wish. They will have no information about your answers. Please indicate how you are most comfortable seeking help.” If the participant reports that they want someone to follow-up with them, their ID will be shared with a non-study staff member at CCIHP. Within three days of the participant’s responses being submitted in REDCap, this staff member will introduce the case and his contact information to an expert (clinical psychologist) who is responsible for supporting participants in the relevant geographic region (North, Central, South). Within 1–3 days after the expert receives the participant’s contact information, the expert will contact the case via phone call to introduce themself and their professional background, and to set an appointment for an online or in-person assessment. The expert will attempt to contact the participant over three days. If unsuccessful, the expert will re-confirm or correct the contact details, and attempt contact again. If successful, the expert will schedule an appointment and complete an assessment, including whether the unmanageable distress was study-related. The expert will make recommendations regarding strategies for intervention, including fee-based and non-fee-based services. If the case refuses support at the time of the expert’s call, the expert will inform the case about available fee- and non-fee-based services. In all cases, the expert will report the general outcomes of their follow-up attempts and whether any unmanageable distress was attributable to the study or the intervention. The study team will report the findings of all adverse events (extreme distress and/or unmanageable distress, regardless of the distress level reported) to the responsible IRBs, independent data safety and monitoring board (DSMB), and study sponsor, in accordance with the timetable required by the National Institutes of Mental Health Reportable Events Policy [55]. In each case, determination about continuation or discontinuation of the program will be made by these parties independently.

### Outcomes {12}

#### Implementation process outcomes (Aim 1).

We will conduct multimethod qualitative research to document all implementation strategies done, to assess modifications to the implementation of GlobalConsent and to implementation plans, and to understand barriers/facilitators to implementation across HIS and LIS groups (Table 4). Twelve group reflections between CCIHP and the study team will be conducted to understand the GlobalConsent implementation process and modifications to the implementation plan in the LIS and HIS groups. Two key informant interviews (KIIs [46]) with each of 30 university leaders (five [56] leaders per university; 15 leaders per IS group; 60 KIIs total) will collect in-depth data from individuals who are knowledgeable about external factors (policies, regulations, funding) and organizational factors (resources, time constraints, institutional climate, leadership support) that may explain modifications to GlobalConsent and implementation plans [46]. A semi-structured interview guide, with open-ended questions and prompts, will guide the KIIs (**Appendix**). Focal topics before implementation will include 1) perceptions about sexual violence among university students and 2) perceptions about the feasibility, acceptability, and suitability of sexual violence prevention programs at universities. Focal topics after implementation will include these topics as well as 1) attitudes of university leaders, faculty, and students about continued implementation of GlobalConsent, 2) barriers and facilitators of future implementations with detailed probes, and 3) external contextual factors that may affect future implementation.

Longitudinal focus group discussions (FGDs) will entail four discussions with each of six groups of 5–8 [57] implementation team members (one group per university; 24 FGDs total). These FGDs will provide detailed, near-real-time data on the project’s dynamic implementation context, including features of the implementation setting; modifications to GlobalConsent or implementation plans; changes in the university, local, regional, or national context that may affect implementation; and team sense-making and learning [58]. The FGD guide [46] includes open-ended questions aligned to FRAME; the rationale, timing, and guidance for each question; and probes about the use of core implementation strategies (**Appendix**). Some questions in the FGD guide are like those in the KII guide to facilitate triangulation of the data during analysis.

A brief checklist will be added at the end of every program module of GlobalConsent (**Appendix**). This checklist will be based on the Modifications and Adaptations Checklist [59, 60], a coding scheme for recording modifications to evidence-based interventions. The checklist will ask participants to self-report any modifications they made to planned use of GlobalConsent. As students cannot skip segments or modules in GlobalConsent, the checklist will focus on the following major modifications: 1) device used to view each module of GlobalConsent, 2) percentage of module watched, 3) number of sessions required to watch the module, 4) whether or not they watched part or all of each module mor than once; 5) ‘drift’ by multi-tasking or doing other things while a module was open, 6) ‘drift’ by stepping away from the computer or mobile device while a module was open, 7) extent of satisfaction with the content of the module, and 8) any comments about the module.

#### Implementation drivers and outcomes (Aim 2).

Table 5 summarizes the constructs to be measured, data sources, study samples, number of assessment points by focal population, and for variables measured in surveys (indicated with a superscript), the number of items per construct. The main constructs to be measured are drawn from implementation science and based on the hypothesized processes and outcomes in the present study. Scales that were created to assess general implementation of evidence-based practices in a medical or social-service setting are adapted to fit the current context of implementation by modifying item wording to focus generally on sexual violence prevention programming and specifically on GlobalConsent in a university context (**Appendix)**.

Demographic implementation drivers that are measured in all focal populations include age in years, sex assigned at birth, gender identity, sexual orientation, and ethnicity. Questions on sex, gender, and sexual orientation are based on recommendations from the National Academies of Sciences, Engineering, and Medicine (NASEM) [61]. Normative implementation drivers include pre-implementation perceptions in all focal populations about sexual violence as a problem to address, perceptions of campus climate, legal knowledge of sexual violence, knowledge about active consent, and rape-myth acceptance. Other implementation drivers include perceptions of implementation 1) leadership, 2) collaboration, and 3) climate.

Implementation outcomes include the 1) perceived acceptability, appropriateness, and feasibility of general sexual violence prevention programming in all focal populations, 2) perceived acceptability, appropriateness, and feasibility specifically of GlobalConsent in all focal populations, 3) implementation adoption among eligible students consenting to take part in GlobalConsent, and 4) implementation penetration among eligible students consenting to take part in GlobalConsent. Intervention adoption and penetration will be measured continuously using records from the collaborative IT company on the implementation of GlobalConsent by tracking the number of eligible male student participants who consent to take part in GlobalConsent (adoption) and who complete the intervention (penetration). Notably, the normative climate among leaders, faculty, and implementation teams at baseline also may change as a result of implementation, so these measures are listed as implementation outcomes.

#### Implementation effectiveness outcomes (Aim 3).

Student-level primary outcomes (sexually violent behavior; prosocial intervening behavior) and student-level secondary outcomes related to cognition/knowledge, attitudes/beliefs, affect, and capacity are summarized in Table 6, and all question sets are provided in English and Vietnamese in the **Appendix**. Table 6 also includes the National Institutes of Health (NIH) common data elements to be collected.

#### Incremental cost effectiveness outcomes (Aim 4).

The primary effectiveness outcomes for the cost-effectiveness analysis will be the frequency of sexually violent behavior and prosocial intervening behavior. The costing items are organized sequentially based on the implementation phases: pre-implementation, implementation, and post-implementation. Each phase includes a detailed list of activities (Tables 2, 3), with costs related to personnel, travel, space, and supplies/equipment collected for each activity. We will estimate the aggregate costs for each university and then divide this aggregate by the total number of participants at that university to determine the cost per participant.

### Participant timeline {13}

**Figure 2.**
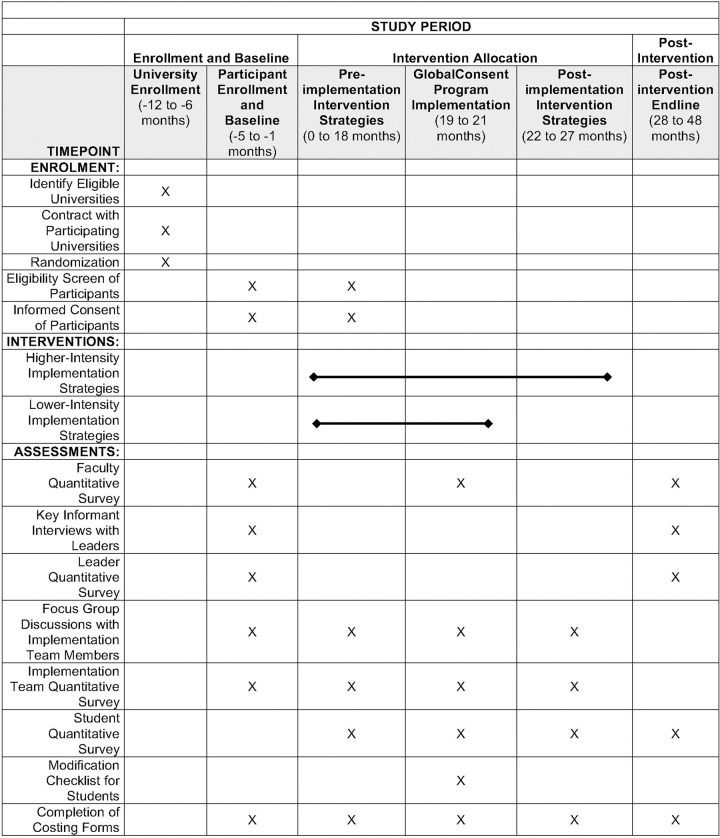
Schedule Of Enrolment, Interventions, and Assessments

Figure 2 summarizes the start dates and end dates for enrolment (X), interventions (implementation activities and delivery of GlobalConsent), and assessments (X) with each focal population in the “high intensity” implementation strategies group.

### Sample size {14}

Focal populations for each of these assessments include university leaders who support the GlobalConsent implementation (n=5 per university, total of 30); members of implementation teams (n≈5 per University, total≈30 members); fulltime permanent faculty at each university (estimated mean 589 per university, range 186–1038, total 3,532; based on official figures for 2021), and male student participants in GlobalConsent (estimated mean n=796 per university, range 449–1202, total=4,776 18–24 year-old, first-year undergraduate, heterosexual or bisexual men, who are attracted to women; based on official enrolment figures for 2021). We will assume 80% participation and 90% retention in each focal population.

### Recruitment {15}

#### University leaders (Aim 1).

University leaders will be identified purposively via discussions between CCIHP key personnel and implementation team focal persons. Eligible participants will be informed during the online informed consent process that they will be compensated $10 for completing each of two online surveys and $20 for completing each key informant interview (KII), for a total compensation of $60 for completing all assessments.

#### Implementation team members (Aims 1, 2, and 4).

Implementation team supervisors and team members will be identified purposively via discussions between CCIHP key personnel and the identified focal implementation team member in each university and finally approved by the leaders of that university. Eligible implementation team members will be informed during the online informed consent process that they will be compensated $10 for completing each of four online surveys and $16 for participating in each focus group discussion (FGD), for a total compensation of $104 for completing all assessments.

#### University faculty (Aim 2).

Each university will submit to non-study staff the list of all permanent, fulltime faculty (lecturers) at their university, with professional contact details. All eligible lecturers will receive an email invitation from staff within the university introducing the study and inviting their participation. Two days later, each faculty member will receive a secure REDCap link to the informed consent form. While completing the online consent form, faculty will have an opportunity to call a non-study staff member to answer any questions about the study, as needed, before consent and participation. Faculty will be informed during the consent process that they will receive $10 for completing each online survey. If consent is confirmed, the faculty member will be directed immediately to the online REDCap survey. Any faculty member who has not completed the online survey will receive up to five automated reminders from REDCap at 2.5-day intervals over two weeks. In addition, on day 8 and 15 of the survey period, all faculty will receive a reminder invitation from within their university to complete the survey. On day 22 and 29 of the survey period, any faculty member who has not completed the survey will receive additional reminder invitations. During the two weeks following day 15, any faculty member who still has not completed the survey will receive up to two standard SMS text reminders with their unique survey link encouraging them to complete the survey. If, after four weeks, faculty have not yet completed the faculty survey, they will receive a standard follow-up reminder and unique survey links via Zalo and a call via Zalo, an encrypted Vietnamese communication application similar to Whatsapp. Once this protocol is completed, implementation teams will send a general reminder through informal faculty networks at their universities for faculty to complete the survey.

#### First-year undergraduate male students (Aims 1 and 3).

All eligible students will be invited to participate in 6 six-monthly surveys and the GlobalConsent program. For the LIS and HIS groups, implementation team members will send a standard email invitation to all eligible men with a description of the GlobalConsent program, a description of the study, and an invitation to participate. They will separately receive a secure REDCap link to the online informed consent form, and if completed, the online survey (Table 6). For the HIS group, internal facilitators will organize an orientation session for all eligible male students to describe these elements in person and to answer any questions about participation. All eligible participants will be informed during the online informed consent process that they will be compensated $5.50 in average for completing each survey assessment, for a total compensation of $33 for completing all six assessments. All eligible participants also will be informed during the online informed consent process that they will be eligible to enroll in a lottery to receive a prize (e.g., smartphone) after completing three survey assessments, and that they will be eligible to enroll in a second lottery to receive a prize (e.g., smartphone) after completing six survey assessments. All eligible participants in the HIS group will be informed about this compensation schedule during the recruitment orientation meeting.

## Assignment of interventions: allocation

### Sequence generation {16a}

All universities in the sample are broadly matched on programs of study (health sciences) and are pair-matched on region (North, Central, South). Within matched pairs, universities will be randomized using a computer random number generator to receive ‘lower intensity’ (LIS) or ‘higher intensity’ (HIS) implementation strategies to deliver GlobalConsent to eligible undergraduate men.

### Concealment mechanism {16b}

The team will separate the act of randomization from the recruitment of participants as follows. First, CCIHP will be responsible for completing the randomization of university to IS groups. Second, a staff member at each participating university will be responsible for recruitment strategies within their respective university. Third, Emory study staff will pre-program the REDCap data system to send automated reminders to participate in each study wave, at the schedules described previously.

### Implementation {16c}

Non-study staff at CCIHP will generate the allocation sequence, randomly assign universities to study arms, and upload contact lists for eligible participants in each focal population into REDCap. Formal invitations to participate will be sent automatically via the customized REDCap data system, or for university leaders, by the staff member at CCIHP conducting KIIs.

## Assignment of interventions: Blinding

### Who will be blinded {17a}

Implementation teams at each university will be blinded to the bundle of implementation strategies to which they are being compared. Students will be blinded to the implementation strategies being implemented at their and other universities, except for the strategies to which they are directly exposed. Study team members at Emory University and Georgia State University will be blinded to the assignments of universities until analyses are completed by using a non-ordered numerical assignment to university and study arm in the REDCap database. Members of the data safety and monitoring board will be blinded to implementation strategy assignments until they request to be unblinded.

### Procedure for unblinding if needed {17b}

Unblinding of implementation strategies assignments may occur before study completion if the Institutional Review Boards, Data Safety and Monitoring Board, or study sponsor deem that unblinding is necessary. Otherwise, unblinding of the study team members will occur upon completion of aims-specific analyses.

## Data collection and management

### Plans for assessment and collection of outcomes {18a}

#### Group reflections with CCIHP.

Each of the 12 group reflections with CCIHP (Table 4) will be recorded, transcribed, and translated. Session transcriptions and team reflection notes will be analyzed on an on-going basis to understand modifications to planned external training and support provided by CCIHP.

#### Key informant interviews (KIIs) with university leaders.

The KII guide will be drafted in English, translated into Vietnamese, piloted, revised, and back-translated into English to confirm consistency of the translation with the original intended meaning. Masters- or PhD-level social scientists at CCIHP with expertise in qualitative research and stakeholder interviews on sexual violence will conduct the interviews. Interviews will occur in the pre-implementation phase, before any educational outreach by CCIHP, and after implementation to assess views and practices related to sustainment. All interviews will occur by audio-conference call. Interviews will be recorded, professionally transcribed, and translated into English. All interviewers will keep a diary in which they will take field notes after each KII [77, 78].

#### Focus group discussions with implementation teams.

The FGD guide will be finalized in English, translated into Vietnamese, piloted, revised, and back-translated into English to confirm consistency of the translation with the original intended meaning. Similar staff at CCIHP will facilitate the FGDs with implementation teams. Discussions will be structured as 45–60-minute in-person discussions [79] held before the start of implementation training and then immediately before, during, and after implementation of GlobalConsent. FGDs will occur on-site at universities. Interviews will be recorded, professionally transcribed, and translated into English. All facilitators will take field notes after each FGD [77, 78].

#### Survey-based data collection (Aim 1 checklist; Aims 2–3).

Survey-based data from all target populations will be collected via REDCap [49], a HIPAA-compliant, web-based data system that allows for the secure collection, transfer, storage, and analysis of study data. Customized surveys will be piloted with individuals like the focal populations but at non-study universities to gauge acceptability, inform final revisions, and assess timings to minimize respondent burden (e.g., 10–15 minutes for each leader, faculty, and implementation team survey; 16 minutes for each student survey). The survey implementation protocol described, above, under “Recruitment” will be followed for at each survey wave for faculty, implementation teams, and students to maximize their participation at baseline (~80%) and retention at endline (~90%), based on prior experience surveying students in Vietnam [51].

Measurement time points will vary for each target sample, according to the project timeline (Figure 2). Leaders will be assessed twice, once before and once after implementation. Implementation teams will be assessed quantitatively at four time points: once before the start of any implementation activities, and once immediately before, during, and after implementation of GlobalConsent. Other data on the implementation will come from administrative records collected by the IT company that is responsible for delivering GlobalConsent. Faculty will complete one assessment before implementation (Year 1) and two after the planned 12-week delivery period of GlobalConsent (Years 3, 5). Three surveys will be administered to students at six-month intervals before the planned 12-week delivery period of GlobalConsent, and three six-monthly surveys will be administered at the same interval after implementation, for a total of six assessments. At each occasion, we will send to students’ smartphones an encrypted link to a REDCap survey with ~189 questions measuring two primary behavioral outcomes and seven cognitive/knowledge, attitudinal/belief, affective, and capacity-related secondary outcomes validated in the efficacy trial [33, 50, 51] (Table 6). Secondary outcomes that are included align with our theory of change (Figure 1) and showed evidence of mediation in the trial [50]. A short modification checklist of 5–10 questions will be administered at the end of each GlobalConsent program module to understand participants’ engagement and satisfaction with the module (Table 4).

#### Costing surveys (Aim 4).

Cost elements for activities involving CCIHP staff and university implementation team members are detailed in the **Appendix**. The costing items for CCIHP should be completed monthly for each CCIHP staff member involved in the GlobalConsent intervention activities. For university implementation teams, costing items should be completed monthly outside the 12-week program implementation period and weekly during the 12-week program implementation period for each team member involved. CCIHP will collect all costing forms monthly outside the 12-week program implementation period and weekly during the 12-week program implementation period, de-identify the data, and then send the forms to the health economist for cleaning and analysis.

### Plans to promote participant retention and complete follow-up {18b}

To maximize retention across occasions, we will conduct brief cognitive interviews and pilot all REDCap surveys in similar populations at other universities in Vietnam. These steps will ensure comprehension of the questions as intended, acceptability of the questions, and appropriate length to minimize participant burden. Implementation teams and the REDCap application will administer a standard protocol of internal and automated reminders to complete each survey. To clarify institutional support and to encourage participation, leaders and/or implementation team members at each participating university will send invitation letters via email prior to survey launch. After the initial survey launch, a standard protocol of follow-up communications will be implemented for all survey waves with faculty, students, and implementation team members to maximize enrollment and retention. University leaders will receive a survey link from the CCIHP study member who conducts their KII, who will confirm individually that they have completed the survey. In addition to implementing a systematic protocol for outreach, participants who complete at least part of each survey will be compensated for their time. Also, for students, we will offer options to enter a lottery to win prizes (a smartphone) after completing all pre-implementation surveys and then all post-implementation surveys.

### Data management {19}

#### Qualitative data (reflections, KIIs, FGDs, field notes for Aim 1).

KIIs and FGDs will be digitally recorded, and all recordings will be numerically labelled. Digital files will be transcribed verbatim, and all Vietnamese transcripts will be fully de-identified, with participants identified numerically or by pseudonym. De-identified Vietnamese transcripts will be translated into English. One CCIHP non-study staff member will check one random segment of each digital file against the transcription and one random segment of each transcription file against the translation to ensure accuracy and/or to make any corrections. Digital files and de-identified transcribed and translated files will be uploaded to separate folders maintained on a HIPAA-compliant, secure network drive maintained by Emory University. Each folder will be accessible only to selected study staff for the purposes of data management and/or analysis. Upon completion of the analyses or within a suitable timeframe, digital files will be destroyed to protect the confidentiality of study participants.

#### Quantitative data (Aim 1 Checklist, Aims 2–3).

The REDCap data system will be used to facilitate secure data collection, transfer, processing, and storage of quantitative data. Data-collection modules for each wave will be built in a REDCap project and self-administered on personal devices to ensure flexibility and privacy. All required fields (e.g., documentation of consent) are specified as such, and participants cannot continue with the survey until required responses are completed. Allowable response options for each question are programmed into the data system, and participants are asked to correct or to confirm any missing or out-of-range responses. Each page of the application includes a check at the bottom to ensure that participants correct or confirm any missing responses to questions on the page. The web application then transmits data entered by each participant through an encrypted network connection to a centralized database running on a secure network and infrastructure maintained by Emory University, ensuring secure electronic data movement.

Each participant is assigned a unique random number identifying him/her across study waves and REDCap projects. All identifiable data (names, contact info, etc.) and randomized ‘lower-intensity’ or ‘higher-intensity’ study arm assignment will be stored with the unique ID and accessible only to non-study staff. The Emory and Georgia State study teams will receive randomization data only after completing the main study analyses but will not have access to identifiers in the REDCap project. CCIHP and Emory staff will create and run applications for more refined range and consistency checks in the centralized database. Systematic data collection errors for each wave of data collection will be identified, resolved, and documented using the logging application in REDCap. For analyses done with external statistical software, de-identified data will be extracted and held on HIPAA-compliant, secure networks and computing workstations.

#### Cost data (Aim 4).

The completed costing forms will be checked for consistency and missing information upon receipt. If any inconsistencies or missing information are found, the forms will be returned to CCIHP or the specific university via CCIHP for correction. Once the forms are verified as complete, they will be stored with a unique university ID and personnel ID in a folder accessible only to the health economist.

### Confidentiality {27}

#### Qualitative data (reflections, KIIs, FGDs, field notes for Aim 1).

No identifying information will be recorded on the KII guide or FGD guide. All digital files will be labelled numerically. All Vietnamese transcripts will be fully de-identified, with participants identified numerically or by pseudonym. All translation files, likewise, will be fully de-identified. Digital files and fully de-identified transcription and translation files will be uploaded to separate folders on a HIPAA-compliant, secure network drive maintained by Emory University. Digital files will be accessible only to selected CCIHP staff for quality management. De-identified transcription and translation files will be accessible only to selected study staff for the purposes of analysis. Upon completion of the analyses or within a suitable timeframe, digital audio files will be destroyed to protect the confidentiality of study participants.

#### Online quantitative survey data (Aim 1 checklist, Aims 2–3).

Procedures to minimize loss of confidentiality include the encrypted entry, transfer, and storage of data collected via REDCap. Collected data also will be identified only by unique, random identification numbers with no personal identifiers. Personal identifiers, for the purposes of recontacting participants for follow-up data-collection waves, will be accessible only to non-study-team members. Confidential data will be maintained on a HIPAA-compliant, secure network drive with user-defined access to selected study team members. Data that are extracted for analysis using other statistical software will be analyzed on password protected computing systems in locked offices. Quantitative study data will be presented in aggregate form only, and participating universities will not be named in the dissemination of study findings.

#### Cost data (Aim 4).

The costing data will be presented at an aggregate level, comparing the HIS group to the LIS group. Participating universities will not be identified in the dissemination of study findings.

### Plans for collection, laboratory evaluation and storage of biological specimens for genetic or molecular analysis in this trial/future use {33}

This section is not applicable, as the study team is not collecting biological specimens.

## Statistical methods

### Statistical methods for primary and secondary outcomes {20a}

#### Qualitative data analysis (Aim 1).

Our analysis of qualitative data will be based on FRAME [36] to enable us to characterize and explain contextual facilitators and barriers to the implementation of GlobalConsent as well as planned and unplanned modifications to implementation strategies in the HIS and LIS groups. We will use rapid content analysis [80, 81] and a hybrid inductive-deductive approach [80, 81] to analyze the data. We will transfer all reflection notes, field diaries, and transcripts of KIIs and FGDs into matrices and use matrix analysis methods to examine core FRAME constructs of 1) engagement with the implementation training materials; 2) GlobalConsent delivery practices; 3) program modifications; 4) implementation strategies over time, including the nature of, timing, reasons for, and decision-makers involved in planned or unplanned implementation modifications; and 5) factors in the outer context or internal organizational context that may explain these modifications. Prior constructs and assumptions will be evaluated against the data, and new themes will be incorporated into the matrix coding scheme [82]. Matrices will systematically note the similarities, differences, and patterns in responses across participating universities and implementation-strategies groups, for a comparative synthesis of the findings [83]. Identifying these barriers, facilitators, and modifications, and their potential influence on implementation drivers and outcomes (Aim 2) and effectiveness (Aim 3) by implementation strategies group will provide insights about recommended adaptations to proposed implementation strategy bundles here as well as the time and skills needed to facilitate the implementation of GlobalConsent by operational partners at other universities.

#### Analysis of quantitative data from leaders and implementation teams (Aim 2).

One set of analyses to explore primary hypotheses for Aim 2 will examine changes over time across LIS and HIS groups in implementation drivers and outcomes among university leaders and implementation teams (Table 5). Quantitative analyses will focus on the same measures across implementation groups for comparisons within and across periods. Cronbach’s α will be estimated with the total sample (N≈78) using pre-implementation baseline survey data to confirm the internal consistency of each scale. Quantitative responses within item sets will be summed, and scores will be standardized by averaging the items in each scale. We will examine correlations of scale constructs at each time point. Comparisons between HIS and LIS groups will be conducted within each time period by target (i.e., faculty, leaders, implementation teams) to examine whether mean differences are statistically significant, at p<.05. We also will examine HIS and LIS group differences in means for implementation drivers (e.g., perceptions of leadership, collaboration, climate) and implementation outcomes (e.g., intervention acceptability, appropriateness, feasibility) at each time point. We will test for differential change over time in mixed models by testing Group x Time interactions with target responses nested within participating university.

#### Statistical power for analyses of leader- and implementation-team data.

We estimated power to test differences in implementation drivers and outcomes of leaders and implementation teams in the HIS and LIS groups. A power analysis was conducted using G*Power [84] and entering a sample size of 78 (39 per group) with four measurement points. Based on repeated measures ANOVA for continuous variables, with alpha set a .05 and assuming a correlation between repeated measurements of .50, the sample of 78 provides excellent power (.95) to detect a small to medium difference (*d* = .33) in differences over time between LIS and HIS groups. We expect little missing data from this collection, but assuming attrition of 20% and a sample size of 62 (N = 31 per group), power still is sufficient (.95) to detect a small-to-medium effect size of *d* = .39.

#### Analysis of quantitative data from the general faculty (Aim 2).

Our analytical approach for general faculty targets will leverage their larger population sizes across universities (Table 1). We will use difference-in-difference (DD) models to assess the effects of being in the HIS group versus being in the LIS group on changes among faculty in norms about sexual violence and awareness of sexual violence as a problem, operationalized using identified scales (Table 5). We will use pre-post implementation data in project Years 1 and 3 to assess short-term effects of exposure to HIS versus LIS on faculty knowledge and attitudes about sexual violence and pre-post implementation data in project Years 1 and 5 to assess longer-term effects of exposure to HIS versus LIS on these outcomes. A basic DD model for our study would take the following form: Y_ijt_ = α_j_ + βP_t_ + γHIS_ij_ + δHIS_ij_*P_t_ + ε_ijt_, where Y_ijt_ is the value of the outcome observed for person i in university j at time t, HIS_ij_ is an indicator of person i in university j being in the HIS (treatment) group (HIS_ij_=1) versus the LIS (comparison) group (HIS_ij_=0), and P reflects the time period (pre=0 vs post=1 implementation of GlobalConsent). The parameter δ is the DD estimator; the point estimate of δ from this model is equivalent to a non-parametric approach that takes the difference in the changes over time between the two implementation strategies groups.

#### Statistical power for analyses of faculty data (Aim 2).

We estimated the power to test the DD models that tested for differences in the knowledge of sexual violence legality and harm between LIS and HIS groups. Using estimates for the mean score measuring the knowledge of sexual violence legality and harm in the GlobalConsent Trial [50, 51], we computed the statistical power with a linear regression model allowing a separate intercept for each university via a simulation. We used the Stata command (ipdpower, model 1, a simulations-based command that calculates power for simple linear regression modeling) to perform the simulation. With a retained sample size of ~2,543 faculty (3,532 X 0.80 participation X 0.90 retention at wave three; ~1,272 in each implementation group), a point estimate of δ of 0.12, alpha level of .05, and 5000 simulations, estimated power was .85.

#### Analysis of quantitative data from students (Aim 3).

To explore the public-health impact of GlobalConsent in the two implementation-strategies groups, we will estimate comparative interrupted time-series (CITS) models for student-level primary outcomes (sexually violent behavior; prosocial intervening behavior) and student-level knowledge, attitudinal, affective, and capacity-related secondary outcomes (Table 6).

As a first step in the analysis, we will inspect pretreatment data closely to select the modeling approach that best fits the data. Given the multi-module nature of GlobalConsent and evidence from the efficacy trial [51], we expect to see immediate level changes (improvements) in all outcomes at the first post-test survey and slope changes for all outcomes, as cognitive, attitudinal, affective, and behavioral change attenuate *partially but not entirely* [51]. We expect to see greater immediate improvement and less attenuation in the HIS group than the LIS group. A basic CITS model for the frequency of men’s sexually violent behavior may take the following form: log(E(Y_ijt_)) = B_0_ + B_1_T_t_ + B_2_Z_j_ + B_3_P_t_ + B_4_T_t_*HIS_ij_ + B_5_P_t_*HIS_ij_ + v_ij_, where Y_t_ is the outcome for the i^th^ participant for the j^th^ university at time t, B_0_ is a constant term showing the average frequency of sexually violent behavior in the reference university before implementation; Z_j_ is a vector of university dummies to allow a separate intercept for each university; T_t_ is the time elapsed since study start, where t=1,…,10 quarters; B_1_ is the pre-implementation trend in the comparison (LIS) group, and Β_1_ + B_4_ is the pre-implementation trend in the HIS group; P_t_ is a vector of indicators for each post-implementation time period; B_3_ is the level change in the reference university in the post-implementation period; v_ij_ is the random effect for the i^th^ participant at university j which would allow random subject-to-subject variation in the intercepts at each university. The difference in the actual post-implementation performance from the projected post-implementation performance in the HIS group, less this same difference in the LIS group, is the estimate of HIS effects (B_5_). This formulation assumes that all universities in the HIS group share the same trend, and all universities in the LIS group share the same trend (though possibly different from the HIS trend). This assumption could be relaxed by modeling the trends as random effects. To assess whether the slope of performance changes after implementation, we may code P_t_ as a dichotomous variable that equals 0 in the pre-implementation period and 1 in the post-implementation period. The change in slope can then be estimated by adding a three-way interaction between the higher-intensity implementation indicator (HIS_ij_), post-implementation indicator (P_t_), and linear time trend (T_t_). In this formulation, P_t_ would be coded to be centered on the introduction of implementation, so B_5_ is interpreted as the immediate shift in outcomes following implementation. Inclusion of this interaction would allow implementation effects to grow or decline over time. A similar CITS model will be applied to investigate the impact of GlobalConsent on the incidents of men’s prosocial intervening behavior in the two implementation-strategies groups.

#### Power for analyses of student data (Aim 3).

We estimated the power to test models that tested for differences in the frequency of sexually violent behavior and prosocial intervening behavior between LIS and HIS groups. Using estimates for the mean frequency of sexually violent acts in the GlobalConsent Trial [51], we computed the statistical power with a generalized linear mixed model with a random intercept for repeated count measures via a simulation. We used the Stata command (ipdpower, model 2, a simulations-based command that calculates power for mixed-effects modeling with random effects for intercept) to perform the simulation. With a retained sample size of ~3,439 men (4,776 X 0.80 participation X 0.90 retention the final wave; ~1,719 in each implementation group), six counts for the frequency of sexually violent acts, an incidence rate ratio of 0.825 (0.65+(1–0.65)/2) for the rate of change after implementation of GlobalConsent for the HIS group relative to the LIS group, alpha level of .05, and 5000 simulations, estimated power was .91.

#### Analytical challenges and solutions.

##### Leader and implementation team analysis:

A primary challenge for this analysis is the non-random selection of the sample and relatively small sample sizes across the LIS and HIS groups. These limitations require the analyses to be largely descriptive and inferences to be restricted to the samples rather than to the university populations from which the samples are drawn. Still, our mixed-methods study design will allow us to triangulate findings from the qualitative and quantitative data collected from the university leaders and implementation teams, and both sets of findings with those from university faculty. This multi-method, multi-sample approach will permit a more nuanced understanding of the institutional changes that may be underway and that may facilitate or inhibit the implementation of GlobalConsent and/or planned implementation strategies. Thus, all findings from our approaches to Aims 1 and 2 will inform interpretation of the results for the implementation effectiveness assessment (Aim 3).

##### University faculty analysis.

DD methods provide unbiased effect estimates if the trend over time would have been the same between the treatment (HIS) and comparison (LIS) groups in the absence of the more intense elements of HIS implementation. However, selection bias may arise if the composition of these groups changes over time, such that the faculty population at participating universities changes systematically, for example, through substantial turnover or consolidation. One approach to address this issue is to restrict the sample of faculty to those who are available across all three survey waves and study years (1, 3, 5). If high turnover risks a substantial loss of power using this approach, we will consider the use of propensity score weighting to handle this type of confounding across four groups (HIS pre, HIS post, LIS pre, LIS post) [85] Another challenge in this analysis is the cluster-randomized design, where six universities are assigned to HIS or LIS groups. We will assess the robustness of our DD findings across a range of small-sample corrections, and we will report the methods used and findings to ensure transparency and reproducibility [86, 87].

##### Male student analysis.

1) We will include the age-standardized male student population in person period as an offset variable to convert the outcome into a rate and adjust for potential changes in the population over time. 2) We will consider methods to adjust for seasonality [88] or other time-varying confounders [89], such as concurrent training programs or changes in COVID-related conditions that alter opportunities for in-person versus online interactions at participating universities. 3) We will diagnose and address potential issues of over-dispersion [88] and residual auto-correlation [90, 91], and 4) we will conduct sensitivity analyses to test the impact of varying model specifications, such as whether a negative binomial regression model fits the data better than a Poisson regression model or different lags in slope changes for behavior and different impact models (e.g., non-linear trend model, school-year fixed effects model) [88, 89, 92, 93] 5) Another potential concern in CITS analyses of self-reported sexually violent behavior is systematic biases in reporting, including over time due to the repeated measurement of this sensitive behavior. Increased under-reporting over time could lead to falsely attributing declines in sexually violent behavior to the implementation of GlobalConsent. However, increased under-reporting due simply to the repeated measurement of sensitive behavior should not differ across LIS and HIS groups, such that estimates of differences in implementation effectiveness across groups should not be biased. Also, a review of survey experiments suggests little difference in reports of frequency of sensitive behaviors using standard interview methods versus month-by-month reporting [94]. Still, we will use various strategies shown to improve the reporting of potentially sensitive behaviors, including computer-assisted self-interview, assurances of anonymity, the choice for men to complete surveys in a private location of their choosing, and the use of multiple response options to capture the frequency of reported behaviors [94].

#### Incremental cost-effectiveness analysis (Aim 4).

The cost-effectiveness analysis will be conducted from the payer’s perspective. Program costs will be calculated using a micro-costing approach, multiplying resource use by unit costs. Costs will be divided into the following two components based on the process necessary to set up and deliver the GlobalConsent program: 1) set-up costs (e.g., initial training costs, and set-up before the start of the program), and 2) program delivery costs (e.g., session time, preparation time, administrative costs, and materials/supplies). Cost data will be cleaned and used to estimate the total costs, set-up costs and program delivery costs per participant for each of the two groups (HIS vs LIS).

Net costs (the net increase in costs from the HIS vs LIS) and net effectiveness (the difference in the actual post-implementation performance from the projected post-implementation performance in the HIS group, less this same difference in the LIS group) will be used to calculate an incremental cost-effectiveness ratio (ICER) (costs per additional incident of sexually violent behavior averted or costs per additional incident of prosocial intervening behavior increased). Bootstrapping techniques will be used to conduct uncertainty analyses to assess variability in our findings from potential sampling bias.

### Interim analyses {21b}

The team will undertake basic descriptive analyses of baseline data from each target sample. The team also will undertake psychometric analysis (exploratory factor analysis, confirmatory factor analysis, and multiple-group confirmatory factor analysis) of each scale-related item set to assess the measurement invariance if item sets across universities, IS groups, and study wave. The study team will present interim results to the study’s Data Safety and Monitoring Board (DSMB) biannually during data collection, and as needed, to the DSMB, Emory IRB, and sponsor if a participant reports unmanageable distress (see Composition of the data monitoring committee, its role, and reporting structure, below). The DSMB and Emory IRB will make independent determinations about the need to terminate the trial, which will be shared with the sponsor for review and a final decision.

### Methods for additional analyses (e.g. subgroup analyses) {20b}

Each participating university will be invited to propose subgroup analyses of the data collected at their respective university. Proposals for these analyses will be submitted to the SCALE Steering Committee and reviewed on a rolling basis (**Appendix**).

### Methods in analysis to handle protocol non-adherence and any statistical methods to handle missing data {20c}

To address protocol non-adherence, we will evaluate the balance between study arms (HIS and LIS groups) based on student responses regarding any modifications to program delivery. If necessary, we will adjust for these modifications in our analysis to reduce potential bias. Moreover, we will conduct a per-protocol analysis, including only those participants who adhered to the protocol. Comparing the results of these analyses with analyses of all participants will provide insights into the impact of non-adherence.

Inadvertent missingness will be minimized at the data collection stage by pre-programming the REDCap data system to inform the participant of any missing responses and to invite them to complete their responses before proceeding to the next survey page. Sensitivity analyses will also be conducted, with missing data imputed under the assumption of missingness at random.

### Plans to give access to the full protocol, participant level-data and statistical code {31c}

The full protocol here, including study forms, will be made available through peer-reviewed publication. Participant-level data will be made available through the National Institute of Mental Health Data Archive (NDA) and Emory dataverse—Emory’s open-data repository—to support the preservation, discoverability, and accessibility of data that team members in this project produce. At the project’s completion, upon publishing the main findings, we will make study documentation, data dictionaries, and the final, cleaned, recoded, and de-identified data available through the NDA and Emory’s dataverse. We will develop a formal data-sharing agreement between key personnel at Emory University, the Center for Creative Initiatives in Health and Population (CCIHP), Georgia State University (GSU), and participating universities. This data sharing plan will describe the subsets of data to be made available to participating universities during the project period (**Appendix**). The data sharing agreement with participating universities will provide standard procedures for applications to use the data and project guidelines for publication. Statistical code for analyses will be made available upon reasonable request to the corresponding author of project-related publications.

## Oversight and monitoring

### Composition of the coordinating centre and trial steering committee {5d}

The overall structure of the study team will include a central Steering Committee comprised of key personnel, each of whom is responsible for one or more specific aims and/or local imple-mentation of the project (**Figure 3**). The principal investigator (PI) at Emory University will chair the Steering Committee and will provide leadership for Specific Aims 1 and 3, related to implementation fidelity and effectiveness. The site-PIs at CCIHP and at Georgia State University will provide leadership for Specific Aim 2 on implementation drivers and outcomes. The health economist co-investigator at Georgia State University will provide leadership for Specific Aim 4 on cost-effectiveness and will provide overall statistical guidance to the study team. The site-PI and co-investigator at CCIHP will provide input on the research design and will lead external implementation support to participating universities in Vietnam. The IT company contracted in Vietnam will deliver the GlobalConsent program to participating students, will send reminders to complete each module, and will track adherence metrics at the student participant level. The Steering Committee will meet weekly to design, implement, and evaluate this initiative. A project coordinator / data analyst at Emory University will support the Steering Committee and will receive opportunities for professional development throughout the initiative. Pre-doctoral students at Emory University and Georgia State University will support all aspects of the research, from design to implementation and analysis and will receive capacity strengthening in research and opportunities for professional development.

### Composition of the data monitoring committee, its role and reporting structure {21a}

#### Roles and membership.

An independent Data and Safety Monitoring Board (DSMB) will include three experts in 1) implementation science, 2) sexual violence prevention, and 3) biostatistics. This DSMB is charged with reviewing study data for quality and integrity, adherence to the protocol, participant safety, study conduct and progress, and making determinations regarding study continuations, modifications, and suspensions/terminations. The monitoring responsibilities of the DSMB will enhance, but will not replace, the monitoring responsibilities of the Principal Investigator (PI) and the IRBs for this project. The PI and study team retain responsibility for real-time management of the study.

#### Independence of DSMB members.

DSMB members will be independent from any professional or financial conflict of interest (COI) with the research project and/or study investigators. Independence ensures that competing interests do not unduly influence the DSMB and supports objectivity that enhances the safety of participants and the integrity of the trial data. Potential DSMB members will provide the NIMH with qualifications and a COI statement indicating that members have no direct involvement with the study or COI with the investigators conducting the study. DSMB members may be affiliated with the investigator’s institution or other participating sites, but cannot be a scientific collaborator or co-author, supervisor, mentor/mentee, subordinate of the investigators, or a member of the investigator’s institutional department within the last three years. DSMB fees will be provided, per NIH and Institutional Policies.

#### Responsibilities and review.

The DSMB will review the DSMP and study protocol before the first participant’s enrollment to establish a charter that clarifies what data points will be monitored, how they will be monitored, and the monitoring schedule. The DSMB review will include, at a minimum: enrollment data, safety data, and data integrity. As this study is blinded, the DSMB may be blinded or unblinded to the intervention assignment but will be able to be unblinded if needed.

#### Metrics for review.

The study team will provide descriptive statistics for the DSMB’s review at each of its meetings. Descriptives will cover questions from the following survey modules for male students and will include information about missingness/non-response.

1. Demographics (once at baseline)
2. Knowledge about sexual violence (from each survey wave)
3. Knowledge about sexual consent (from each survey wave)
4. Rape Myths (from each survey wave)
5. Skills to engage in healthy communication (from each survey wave)
6. Rape empathy (from each survey wave)
7. Readiness to intervene (from each survey wave)
8. Bystander intervention strategies (from each survey wave)
9. Sexual Experiences (from each survey wave)
10. Single question on distress (SRQ-20 item 6)

In addition to the above enrollment and safety data, the study team will provide data to the DSMB on the following study-related variables from male students:

1. Participation rates in the survey, by university
2. Retention rates in the survey at each wave, by university
3. Participation rates in the program, by university
4. Retention rates in the program, by university

#### Review schedule and monitoring reports.

The DSMB meeting/review schedule will be commensurate with the level of risk involved with the study but will occur no less than once per year. Additional reports may be requested, and additional meetings may be called as needed to address issues regarding participant safety. Members of the investigative team may be present for the open portion of a DSMB meeting, but not for the closed deliberations or the vote to recommend continuation, suspension, or termination of the study. The DSMB will issue a monitoring report to the PI after each review/meeting. This report will include any significant actions taken and the final recommendation(s) regarding the study’s continuation. These reports will be submitted to National Institute of Mental Health (NIMH) program staff in the annual progress report. The planned frequency of meetings is annually in 2023, 2026, 2027 before and after the period of data collection and program implementation; and twice annually in 2024 and 2025, during the period of data collection and program implementation.

### Adverse event reporting and harms {22}

For this study, standard adverse-event definitions are used. An adverse event (AE) refers to any unfavorable and unintended sign (including distress), symptom, or disease temporally associated with the use of a medical treatment or procedure, regardless of whether it is considered related to participation in the GlobalConsent program. A serious adverse event (SAE) is any AE that is life-threatening or results in death, an event requiring inpatient hospitalization or prolongation of existing hospitalization, or persistent or significant disability/incapacity.

Adverse events are graded as mild, moderate, or severe. A mild AE is an experience that is transient and requires no special treatment or intervention. The experience does not generally interfere with usual daily activities. This experience includes transient laboratory test alterations. A moderate AE is an experience that is alleviated with simple therapeutic treatments. The experience impacts usual daily activities. This experience includes laboratory test alterations indicating injury, but without long-term risk. A severe AE is an experience that requires therapeutic intervention. The experience interrupts usual daily activities. If hospitalization (or prolongation of hospitalization) is required for treatment, it becomes a SAE.

The study uses a standard adverse-event attribution scale. Not related means that the AE clearly is not related to the study procedures (i.e., another cause of the event is most plausible and/or a clinically plausible temporal sequence is inconsistent with the onset of the event). Possibly related means that an event that follows a reasonable temporal sequence from the initiation of study procedures, but that could readily have been produced by several other factors. Related means that the AE is clearly related to the study procedures.

A comprehensive list of resources will be made available to every participant toward the end of every survey to ensure that all participants have access to confidential care. In addition, adverse events among students who are receiving the GlobalConsent program will be identified with a series of self-report questions at the end of every survey. These questions will identify the level of distress the participant is experiencing in the moment and its self-reported manageability. Any participant who reports extreme distress (level 10 on a scale of 1–10) or any level of distress that they identify as “not at all manageable” will be given a contact number for an experienced professional for immediate support. Such participants also will be invited to have a non-study staff member connect them with an experienced professional for a confidential assessment and then appropriate referrals.

### Management of risks to participants

#### Expected adverse events.

Expected adverse events associated with the use of the web-based sexual violence prevention program include: 1) mild distress from participation and potential recall of incidents of sexual violence and 2) loss of confidentiality related to sexually violent behavior reported by a participant.

#### Adverse Events Management.

All adverse events will be reported to the Institutional Review Boards of Emory University and the Hanoi University of Public Health our DSMB, and our sponsor following the National Institutes of Mental Health (NIMH) Reportable Events Policy (https://www.nimh.nih.gov/funding/clinical-research/nimh-reportable-events-policy). The trial IRBs and DSMB will make independent determinations about the AE and will make recommendations regarding next steps in the study and data collection. The study team will refer any participant who experiences a stress-related adverse event to confidential counselling in Vietnam, following procedures already described, above.

### Frequency and plans for auditing trial conduct {23}

The study does not have plans for a formal, independent audit; however, several independent bodies will conducting regular reviews of the trials conduct and quality. First, the Emory IRB will provide independent, on-going oversight of implementation of the IRB-approved study protocol through its annual review. A logging system ensures real-time documentation of all communications between study staff and the Emory IRB, including initial protocol submission; IRB review and approval; and submission, review, and approval of all amendments to the IRB approved study protocol. Second, an independent Data Safety and Monitoring Board (DSMB) will review data quality and study progress, will make assessments regarding any reported adverse events, will ensure that all participants receive appropriate support and care, and will submit separate determinations regarding study continuation or discontinuation during the annual report to the NIMH. The DSMB may request documentation of consent and documentation of data management. The REDCap data system is HIPAA-compliant, securely stores all study data, and includes an automated logging function that documents any modification that participants and user-defined study staff make to study data in REDCap. The project officer at NIMH will review all DSMB reports to ensure compliance. Emory requires that all investigators complete an annual financial interest disclosure.

### Plans for communicating important protocol amendments to relevant parties (e.g. trial participants, ethical committees) {25}

Important protocol amendments will be submitted to the study IRBs, the DSMB, and the study sponsor as they arise, so all entitles have an opportunity to offer feedback. Trial modifications also will be recorded on the trial registration site of clinicaltrials.gov.

### Dissemination plans {31a}

The final data from this study will include the following from six universities in Vietnam: two rounds of key informant interviews and surveys with 30 university leaders (60 interviews); four rounds of focus group discussions and surveys with six implementation teams (with approximately 8 members each); three rounds of climate surveys with university faculty; and six six-monthly surveys with male university students. We will work with the NIMH Data Archive (NDA) and Emory dataverse to support the preservation, discoverability, and accessibility of data that team members in this project produce. At the project’s completion, we will publish the main findings in peer-reviewed journals. We then will make study documentation, data dictionaries, and the final, cleaned, recoded, and de-identified data available through the NDA and Emory’s dataverse. We will develop a formal data-sharing agreement between collaborators at Emory University, the Center for Creative Initiatives in Health and Population (CCIHP), Georgia State University (GSU) and all participating universities. This plan will describe what data will be shared with participating universities during the project period for analysis and publication and the process for requesting project data. The plan will emphasis equity among participating university and appropriate oversight of data use by key personnel.

Aggregate project findings also may be disseminated widely as working papers on institutional websites, presentations at international and regional scientific meetings, dissemination workshops in Vietnam and the United States, and articles in peer-reviewed journals in the social and behavioral sciences and public health. We will host dissemination seminars at participating universities, at which the study findings will be shared and discussed with university leadership, to guide university policy, campus climate surveys, and campus programming to reduce the incidence of campus sexual violence, with robust attention to the experiences of students. As appropriate, we also will engage regional and national officials in dialogue about the findings, to support evidence-based policies that improve the environment for implementing sexual-violence-prevention programs on university campuses in Vietnam.

## Discussion

Our proposed project will be the first to assess two multifaceted implementation strategies to deliver a theoretically grounded, efficacious web-based sexual-violence prevention program to undergraduate men attending six universities across Vietnam. If successful, our multidisciplinary, cross-cultural team will be the first to bring rigorous evidence to university and national leaders of the contextual effectiveness of these strategies for delivering web-based sexual-violence prevention programming to large populations of men in adolescence, a period of heightened risk for sexually violent behavior. Our choice to develop, test, and scale GlobalConsent with universities in Vietnam is strategic, given the scale of sexual violence among young people, rapidly expanding rates of university attendance, and the openness of several university leaders to efficacious programming about sexual violence. Our choice to engage universities across all regions of Vietnam provides a novel test of these implementation strategies in different structural and sociopolitical environments, with promise to advance sexual-violence prevention policies in university systems at regional and national levels. Evidence for the effectiveness and incremental cost-effectiveness of these implementation strategies across regions will pave the way for GlobalConsent to address an important, gendered risk factor for chronic mental, physical, and behavioral health conditions over the life course. Thus, by providing novel evidence about how best to bring GlobalConsent to scale nationally, our team has the potential to reduce gender-related health inequities and to improve quality of life by averting acts of sexual violence that may lead to chronic health conditions over the life course among victims. By partnering with universities engaged in CONVERGE, an on-going violence-prevention training program in Vietnam (D43TW012188), these innovations will be achieved through synergistic investments to strengthen local capacity for implementation research, data harmonization, and stakeholder engagement to manage and to prevent sexually violent behavior in young people.

## Figures and Tables

**Table 1.** Characteristics of Participating Universities in Vietnam

University	Founded	Region	Primary Undergraduate (UG) Programs of Study	Depts	Fulltime Lecturers (Estimate)	2021 1^st^ Year Undergrad Enrollment	2021 1^st^ Year Undergrad Men (Estimate)
1	1979	North	General/Traditional Medicine, Dentistry, Public Health, Nursing, Medical Testing, Pharmacy	72	406	1,249	625
2	1902	North	General/Traditional/Preventive Medicine, Nursing, Medical Lab. Techniques, Public Health, Ophthalmic Refraction, Nutrition	80	1,000	1,200	600
3	1957	Central	General/Traditional/Preventive Medicine, Odonto-Stomatology, Pharmacy, Nursing, Medical Testing, Medical Technology, Public Health, Midwives	30	456	1,599	800
4	1963	Central	General medicine, Pharmacy, Nursing, Medical Testing, Medical Technology, Rehabilitation, Public Health	21	186	897	449
5	1947	South	General/Traditional–Medicine, Pharmacy, Odonto-Stomatology, Nursing and Medical Technology, Public Health, Basic Sciences, Rehabilitation	7 (14/162)	1,038	2,403	1,202
6	1979	South	Medicine, Odonto-stomatolory, Pharmacy, Nursing and Medical Technology, Public Health, Basic Sciences, and Traditional Medicine	56	446	2,200	1,100
Total					3,532	9,548	4,776

Notes. Universities are numbered to protect their privacy. The figures are estimates based on 2021 data.

**Table 2:** “Lower” and “Higher” Intensity Strategies to Implement GlobalConsent with Undergraduate Men, Six Universities in Vietnam

Implementation Strategies (IS), by Cluster	IS Group LIS HIS	Implementation Strategy Description	Timing	Duration	Facilitator	Barriers/Facilitators Identified in Stakeholder Interviews to be Addressed
**1) Develop Interrelationships Between Stakeholders and Engage University Leaders and Faculty**						
**Obtain formal commitment (prework)**	**+ +**	**+ Site-specific invitation** to participate led by CCIHP; institutional letter of support describing project and collaboration mechanisms; formal contracting	B	2 hr	External (CCIHP)	Universities familiar w/external facilitation No sexual violence prevention/response policy Limited funding for university programs
**Provide passive external web-support and educational materials**	**+ +**	**+ Passive access** to web-based educational materials: links to research articles, short videos, 1-page briefs on sexual violence among young people; the protocol and efficacy of GlobalConsent in Vietnam; answers to FAQs	C	0 hr	External (CCIHP)	Limited time for learning, need for flexibility Standard practice in the United States
**Conduct educational outreach to strengthen formal commitment**	**+ +**	**+ Pre-implementation**: One webinar per group to define sexual violence; rates among young people in Vietnam; acute/chronic effects over life course; primary-prevention EBIs; recap of project description and collaboration; share GlobalConsent website for passive access to educational materials	B	2 hr	External (CCIHP)	Sustain initial commitment Address myth that sexual violence is rare Increase openness to address sexual violence, inequitable gender norms, rape myths Webinar feasible regardless of COVID situation
**Inform local opinion leaders**	**++**	**+ Monthly emails** from trained internal facilitators to university leaders with updates on implementation progress **+ Town halls (3)** with the general faculty to define sexual violence; rates in young people; acute/ chronic effects over life course; primary-prevention EBIs	D D	1 hr 3 hr	Internal Facilitator	Limited time for meetings, need for flexibility in modes of communication, build sustained communication channels between trained internal facilitators and university leaders
**Conduct educational outreach to sustain program**	**+**	**+ Post-implementation**: One webinar to share anonymized findings (by IS group); discuss plans for sustainment (including guidance on how to handle reporting of sexual violence in existing university counseling centers)	A	1 hr	CCIHP and Internal Facilitator	Concern findings will harm univ. reputation Univ. have underutilized counseling centers, staff; no process to address sexual violence
**2) Engage Potential Consumers (University-Student Users) of GlobalConsent**						
Prepare consumer to be active participants	**+ +**	**+ Email introduction** to GlobalConsent; process and schedule for delivery; procedures for data collection; consent to participate	B	½ hr 0 hr	Internal Facilitator	Standard practice in US universities
Conduct educational outreach	**+ + +**	**+ Email invitation** to an in-person orientation to GlobalConsent **+ One in-person orientation**: intro to GlobalConsent; process of delivery; data collection procedures; Q and A; consent to participate **+ Monthly 1-hr learning sessions** to provide technical assistance (TA) with initial and/or sustained access to the web-based program	B D	2 hr 3 hr	Internal Facilitator	Low awareness among students about SV Students are busy, may be shy to discuss sex with teachers, are likely to seek info separately TA may be needed to access program
Intervene with consumer to enhance adherence	**+ + +**	**+ Email/SMS module completion reminders** every 2 weeks for 12 weeks **++ Email/SMS module completion reminders** every week for 12 weeks	D D	6 × 12 ×	External IT company	(Frequent follow-up, reminders) may be needed, given students time commitments
Alter incentives/ Increase demand	**+**	**+ Lottery option** to win prizes upon program completion	D	0 hr	External IT company	Stakeholder engagement, sustainment given students other time commitments

Notes. B=before implementation; D=during implementation; A=after implementation; C=continuous (before, during, after implementation)

**Table 3.** External Training and Support Activities Provided by CCIHP for Implementation Teams Delivering GlobalConsent

Implementation Strategies (IS), by Cluster	IS Group LIS HIS	Implementation Strategy Description	Timing	Duration	Barriers/Facilitators Identified in Stakeholder Interviews to be Addressed
**Provide passive external web-support and educational materials**	**+ +**	**+ Passive access** to web-based educational materials: links to research articles, short videos, 1-page briefs on sexual violence among young people; the protocol and efficacy of GlobalConsent in Vietnam; answers to FAQs	C	0 hr	Limited time for learning, need for flexibility; Standard support available in the United States
**Conduct educational outreach**	**+ +**	**+ In-person technical training (one per group)** on campus-wide implementation of GlobalConsent; **discussion and demonstration** of GlobalConsent program; **standardized implementation manual**	B	3 dy	Siloed depts limit campus-wide programs
**Train for leadership, prepare champions**	**+**	**+ In-person leadership training (one)** to champion GlobalConsent with internal stakeholders (leaders, implementation teams; faculty, students): leadership styles; managing teams; influence without authority; managing conflict; emotional intelligence; negotiation; leading change	B	2 dy	Skills needed to engage diverse internal stakeholders, lead change; enable local facilitators to support program sustainment
**Provide external support, on-going consultation, technical assistance**	**+**	**+ Biweekly (six) 1-hour recorded quality-improvement team webinars** to provide refresher training; assess implementation progress; assess modifications; build peer-network; provide anonymized data on implementation progress for discussion	D	6 hr	Limited time for facilitation, COVID-related dis-ruptions in workplace warrant webinar format Anonymized data due to sensitivity of topic, concerns over university reputation, and belief that sexually violent behavior may be uncommon

Notes. B=before implementation; D=during implementation; A=after implementation; C=continuous (before, during, after implementation)

**Table 4.** Data Collection Methods for Implementation Assessment (Aim 1)

Method	Stakeholder Group	N	# of Rounds	Total
Team reflections with CCIHP	CCIHP (external facilitators)	NA (external)	12	12
Key informant interviews (KIIs)	University leaders	5 per university 15 per IS group 30 total	2	60
Focus group discussions (FGDs)	University implementation teams	1 per university 3 per IS group 6 total	4	24
Modification checklist	First-year male student participants	637 per university 1910 per IS group 3821 total	1	~3821

Note. IS=implementation strategy

**Table 5.** Implementation Outcomes and Potential Drivers Aligned with RE-AIM [24, 25] and Proctor et al [32] for Comparison Across Implementation Strategies Groups

Construct	Measures	# items	Study samples	# waves

**Potential Drivers of Implementation Outcomes**				
Demographics	Common questions: Age, sex assigned at birth, gender, sexual orientation, ethnicity [33, 61]	8–20^a^	Leaders (Administrator)	1
			General faculty	1
			Implementation teams	1
			Students	1

Institutional norms about sexual violence (SV)^b^	Participation in SV programming	3	Leaders	2
	Perceptions of SV at one’s university	3	General faculty	3
	Perceptions of campus climate [62]	6	Implementation teams	4
	Legal Knowledge of SV [33, 63]	12	Students	3B/3A
	Active consent knowledge [33]	12		
	Rape Myth Acceptance [33, 64, 65]	15		

Implementation leadership^b^	Implementation Leadership Scale [66]	13	Implementation teams	4

Implementation collaboration^b^	Cultural Exchange Inventory [67]	7	Implementation teams	4

Implementation climate^b^	Implementation Climate Scale [68]	12	Implementation teams	4

**Implementation Outcomes**				

Intervention acceptability^b^	Acceptability of Intervention [69]	4	Leaders	2
			General faculty	3
			Implementation teams	4
			Students	2B/2A

GlobalConsent acceptability^b^	Acceptability of GlobalConsent (adapted) [69]	4	Leaders	2
			General faculty	3
			Implementation teams	4
			Students	2B/2A

Intervention appropriateness^b^	Intervention Appropriateness Measure [69]	4	Leaders	2
			General faculty	3
			Implementation teams	4
			Students	2B/2A

GlobalConsent appropriateness^b^	GlobalConsent Appropriateness Measure (adapted) [69]	4	Leaders	2
			General faculty	3
			Implementation teams	4
			Students	2B/2A

Intervention feasibility^b^	Feasibility of Implementation Measure [69]	4	Leaders	2
			General faculty	3
			Implementation teams	4
			Students	2B/2A

GlobalConsent Feasibility^b^	GlobalConsent Feasibility of Implementation Measure (adapted) [69]	4	Leaders	2
			General faculty	3
			Implementation teams	4
			Students	2B/2A

Implementation adoption	Administrative records: # of students consenting to take part in GlobalConsent	N/A	Students	C

Implementation penetration	Administrative records: # of students completing GlobalConsent	N/A	Students	C

a Number of demographics questions varies across samples

b Denotes variables derived from surveys with target samples

Notes. B=Before implementation; A=after implementation; C=continuously measured; implementation teams include team supervisors and team members

**Table 6.** Primary and Secondary Implementation Effectiveness Outcomes Among Students

Construct	Original Scale	# items, response coding	Example Item
**Primary Behavioral Outcomes**			
Prosocial Intervening Behavior	Bystander Intervening Behavior [20, 33, 50, 70]	7 0–2+	I have told guys not to talk about women in sexually degrading ways.
Sexually Violent Behavior	Sexual Experiences Survey [71]	45 0–3+	I watched someone while they were undressing, were nude, or were having sex, when they did not agree to it.
**Secondary Cognitive/Knowledge Outcomes**			
Sexual Violence Legality and Harm	Legal Knowledge Scale [33, 63]	10 0–2	Taking a sexual photo or video of someone without consent
Active Consent	Sexual Consent Scale [33, 72]	6 0–4	A person can express non-consent for sex at any time during sexual contact.
**Secondary Attitudinal/Belief Outcomes**			
Rejection of Rape Myths	Vietnamese Rape Myth Acceptance Scale [33]^a^	9 0–4	In the majority of rapes, the victim is promiscuous or has a bad reputation.
**Secondary Affective Outcome**			
Empathy for Rape Victims	Rape Empathy Scale [73]^b^	10 0–1	During a trial, I empathize more with the feelings of the rapist than of the victim.
**Secondary Capacity-Related Outcomes**			
Sexual Communic-ation Self-Efficacy	The Sexual Communications Scales [74]	5 0–2	Talking about sex with a dating partner.
Bystander Self-Efficacy	Bystander Efficacy scale [20]	11 0–2	Express your discomfort if a guy makes a joke about a woman’s body.
Bystander Intention to Intervene	Readiness to Intervene [75]	5 0–2	I am planning to learn more about the problem of sexual violence on campus.
**NIH Common Data Elements**			
Generalized Anxiety	GAD-7	7 0–3	Over the last two weeks, how often have you been bothered by trouble relaxing?
Depressive Symptoms	PHQ-9	9 0–3	Over the last two weeks, how often have you been bothered by feeling tired or having little energy?
Cross-cutting Mental Health Domains	DSM-5 Cross-cutting Adults	23 0–4	During the past two weeks, how often have you been bothered by feeling down, depressed, or hopeless?
Difficulties Due to Health Conditions	WHODAS 2.0	12 0–4	In the past 30 days, how much difficulty did you have in your day-to-day work?

a Based on Illinois Rape Myth Acceptance scale [64] and College Date Rape Attitudes & Behaviors Scale [65]

b Based on Deitz et al. (1982) [76]

## Data Availability

The study forms are available in the Appendix of this protocol, and the study data will be made publicly available through the National Institute of Mental Health Data Archive (NDA) and the Emory Dataverse.

## Abbreviations

AE: Adverse Event
AIDS: Acquired Immune Deficiency Syndrome
C: Continuously measured
CCIHP: Center for Creative Initiatives in Health and Population
CITS: Comparative Interrupted Time Series Analysis
DD: Difference-in-difference model
DSMB: Data Safety and Monitoring Board
EPIS: Evidence-based Practice Implementation in public Service sectors
ERIC: Expert Recommendations for Implementing Change
FGD: Focus Group Discussion
FRAME: Framework for Reporting Adaptations and Modifications-Expanded
GSU: Georgia State University
HIS: High-Intensity Implementation Strategies
HIV: Human Immunodeficiency Virus
ICER: Incremental cost-effectiveness ratio I
IRB: Institutional Review Board
IS: Implementation Strategies
IT: Information Technology
KII: Key-Informant Interview
LIS: Low-Intensity Implementation Strategies
LMIC: Low- and Middle-Income Countries
NASEM: National Academies of Science, Engineering, and Medicine
NDA: NIMH Data Archive
NIH: National Institutes of Health
NIMH: National Institutes of Mental Health
RE-AIM: Reach-Effectiveness-Adoption-Implementation-Maintenance
OR: Odds Ratio
PEPFAR: President’s Emergency Plan for AIDS Relief
SAMSHA: Substance Abuse and Mental Health Services
SCALE: Strategies for Implementing GlobalConsent to Prevent Sexual Violence in University Men
SPIRIT: Standard Protocol Items: Recommendations for Interventional Trials
TA: Technical Assistance
TOR: Terms of Reference
US: United States
USAID: United States Agency for International Development
WHO: World Health Organization
